# The complete chloroplast genome sequence of *Flacourtia jangomas*

**DOI:** 10.1080/23802359.2019.1668731

**Published:** 2019-09-24

**Authors:** Xiaoyan Zhang, Shuyu Liu, Yang Tian, Yi Li, Jianguo Zhang, Zhaoshan Wang

**Affiliations:** a State Key Laboratory of Tree Genetics and Breeding, Key Laboratory of Silviculture of the State Forestry Administration, Research Institute of Forestry, Chinese Academy of Forestry, Beijing, China;; b Collaborative Innovation Center of Sustainable, Forestry in Southern China, Nanjing Forestry University, Nanjing, China

**Keywords:** *Flacourtia jangomas*, complete chloroplast genome, phylogenetic analysis

## Abstract

In this study, the complete chloroplast genome sequence of *Flacourtia jangomas* was featured from Illumina pair-end sequencing. In *F. jangomas*, its length was 156,223 bp, consisting of a large single copy (LSC) region of 84,233 bp, two inverted repeat (IR) copies 27,649 bp and a small single copy (SSC) region of 16,693 bp. The entire GC contents were 37%, and in the LSC, SSC, and IR regions were 35, 31, and 42%, respectively. *Flacourtia jangomas* has 130 unique genes, including 86 protein-coding genes, 36 tRNA genes, and 8 rRNA genes. The chloroplast genomes of 12 species of Salicaceae and 1 species of Euphorbiaceae were used to construct the maximum-likelihood phylogenetic tree. It indicated that *F. jangomas* belongs to Salicaceae and is clustered with *Flacourtia indica* as sisters.

## Introduction

*Flacourtia jangomas* (Lour.) Rauschel, a species of *Flacourtia* (Salicaceae), with varying names such as coffee plum, East Indian plum, Indian plum, and Manila cherry among others. It mainly distributed in the southeast of China and Vietnam, which is a shrub or small tree (Ishikawa et al. 2004). As a medicinal plant, it plays an important role in the economy and ecology. But wild *F. jangomas* are facing serious damage because of overexploitation. In this study, we characterized the whole chloroplast genome of *F. jangomas* and comprehended more about genetic information of this species, which can contribute to the conservation, and supply useful help for population genetics studies of *F. jangomas*.

The leaf samples of *F. jangomas* were collected from Jinhong (Yunnan, China; 21°54″N,102°32″E), and stored the voucher specimen (FJ20180622-4) at the herbarium of Research Institute of Forestry, Chinese Academy of Forestry. A modified CTAB method was used to isolate total genomic DNA from silica gel-dried leaves (Doyle and Doyle 1987). The isolated DNA stored in the −80 °C refrigerator at the herbarium of Research Institute of Forestry. Sequencing was carried out on the Illumina HiSeq 2000 platform (Illumina, San Diego, CA). We used MITObim 1.8 (University of Oslo, Oslo, Norway; Kaiseraugst, Switzerland) to package the chloroplast genome (Hahn et al. 2013) and use *Flacourtia indica* (GenBank: MG262341) as the reference. We annotated the complete chloroplast genome using DOGMA software (Wyman et al. 2004) and then submitted by Sequin 15.50 (http://www.ncbi.nlm.nih.gov/Sequin/).

The chloroplast genome of *F. jangomas* (GenBank accession MK572740) has a total length of 156,223 bp and is composed of a large single copy (LSC) region of 84,233 bp, two inverted repeat (IR) copies 27,649 bp and a small single copy (SSC) region of 16,693 bp. The entire GC contents were 37%, and in the LSC, SSC, and IR regions were 35, 31, and 42%, respectively. The proportion of coding sequences including all RNA regions is 59.5%. A total of 130 unique genes, including 86 protein-coding genes, 36 tRNA genes, and 8 rRNA genes. Seventeen genes contain introns, and three of these genes, rps12, and ycf3, clpP exhibit two introns. The 5′-end exon of the rps12 gene was located in the LSC region, and the 3′-end exons, as well as intron of the gene, were situated in the IR region. A total of 19 of these genes were duplicated in the IR regions including 7 tRNAs (trnA-UGC, trnI-CAU, trnI-GAU, trnL-CAA, trnN-GUU, trnR-ACG, and trnV-GAC), 8 protein genes (rps7, rpl2, rpl23, rps12, rps19, ndhB, ycf1, ycf2, and ycf15) and 4 rRNAs (4.5S, 5S, 16S, and 23S rRNA). Also, partial of ycf1 duplicated in IR region is formed as one pseudogene.

**Figure 1.**
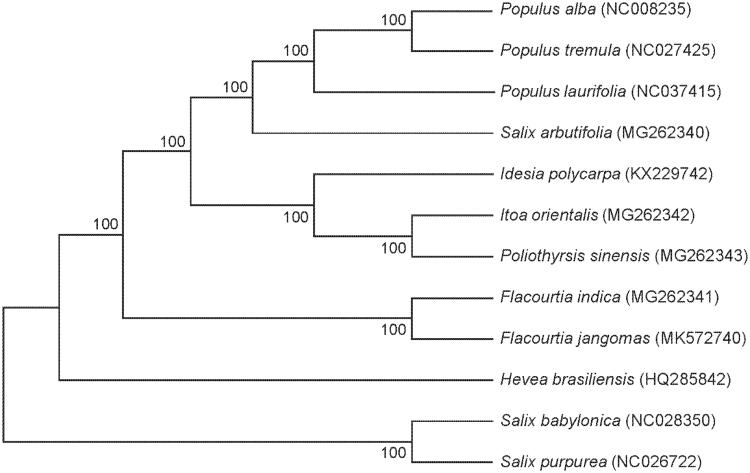
Neighbour-joining (NJ) analysis of *F. jangomas* and other related species based on the complete chloroplast genome sequence. The gene’s accession number is listed in the figure.

We used MEGA 7.0 (Kearse et al. 2012) by the MAFFT (Katoh and Standley 2013) to construct a maximum-likelihood phylogenetic tree (with 1000 bootstrap replicates), and the phylogenetic tree including 10 related species of Salicaceae, and *Hevea brasiliensis* (Euphorbiaceae) as the outgroup. The results meant that *F. jangomas* and *F. indica* were as close as sisters, and suggested that *F. jangomas* belongs to Salicaceae as observed in the phylogenetic tree (Figure 1).

In conclusion, the complete chloroplast genome in this study promotes the phylogenetic studies of the Salicaceae family.
